# Assessment of the nutritional status and dietary intake of women of childbearing age in the city of Comè, Benin

**DOI:** 10.11604/pamj.2022.41.350.33393

**Published:** 2022-04-29

**Authors:** Aymeric Joaquin Darboux, Victoire Agueh, Reynald Yered Santos

**Affiliations:** 1 Community of Practice in Ecohealth for West and Central Africa (COPEH-WCA) & Faculty of Health Sciences, University of Abomey-Calavi, Godomey, Benin,; 2 Faculty of Health Sciences, University of Abomey-Calavi, Godomey, Benin,; 3 Department of Health Promotion, Regional Institute of Public Health Comlan Alfred Quenum, University of Abomey-Calavi, Godomey, Benin

**Keywords:** Women of childbearing age, dietary intake, nutrition, Benin

## Abstract

**Introduction:**

over nutrition and undernutrition problems affect many women of childbearing age in Benin. The inadequacy of the diet is the major reason. The objective of this study is to assess their nutritional status and diet in Comè, a city with the highest proportions of adverse neonatal outcomes in the Mono regional department of Benin in 2015.

**Methods:**

data were collected in June 2017 in a cross-sectional survey with a two-stage random sampling of non-pregnant or lactating women aged 15-49 years. Their sociodemographic characteristics, diet and nutritional status were reported and explored through descriptive and bivariate analysis.

**Results:**

the prevalence of underweight was 9.5% and overweight (overweight and obese) was 31.7%. The dietary diversity score was low for 47.9% of them. Compared to the Recommended Dietary Allowances, the energy and protein intakes were insufficient for 78.7% and 11.8% respectively. None of them had an energy-balanced diet according to the energy distribution between macronutrients after normalization of intakes. The contribution of carbohydrates was high in 99.1%, low in 96.2% for fats and adequate in 60.2% for proteins.

**Conclusion:**

these results reiterate the importance of developing nutritional interventions to improve the nutritional status and the diet of women of childbearing age in Benin. Periodically national food surveys must be conducted to evaluate their real nutritional intakes and promote better nutrition.

## Introduction

Adequate nutrition is a pillar of individual health, especially for women. Women are more likely to suffer from nutritional deficiencies than men in developing countries for reasons related to their reproductive biology, low social status, poverty and lack of education [1]. Studies have shown that a bad nutritional status leads to medical and obstetric complications such as cardiac or respiratory disorders, anemia, premature rupture of membranes, endometritis (endometrial infection), intrauterine fetal growth retardation, spontaneous abortion, prematurity of the child, and low birth weight [2-5]. These complications are known to increase the risk of maternal and neonatal mortality. It was estimated that each year maternal undernutrition contributes to nearly 800,000 neonatal deaths [6]. So the nutritional status of women of childbearing age (WCA) is a key factor in the success of pregnancies, maternal survival, and the health of children at birth. Although WCA are increasingly affected by over nutrition problems such as overweight, obesity, and high blood pressure [7], deficiency nutritional problems such as anemia and chronic energy deficit persist. They are both promoted by poor dietary habits [8,9].

Worldwide, the prevalence of anemia remains high among WCA (32.8%) [10]. In 2016, 613.2 million WCA had anemia of which 35.3 million were pregnant, 1.02 billion women were overweight of which 393.5 had obesity and 153.8 million women were underweighted [10]. In sub-Saharan Africa, evidence indicate that 8.9% of women were underweight and 39.7% overweight or obese [11]. In Benin, 58% of WCA had anemia, 11% were underweight and 26% overweight or obese [12].

In 2015 in the Mono regional department of Benin, the proportion of children born with low birth weight (10.3%) and the ratio of early neonatal deaths (37.1 deaths per 1000 live births) were highest in the city of Comè [13]. The same was true for the proportion of stillbirths and abortions (7.4%) [13]. Given the link between the nutritional status of women of childbearing age and pregnancy outcomes, we suspect that WCA´s dietary intakes are not sufficient to meet their nutritional needs. To our knowledge, no studies have been conducted in this region of Benin to investigate this situation. The present study aims to fill this gap. Its main objective is to assess the nutritional status and diet of WCA in the city of Comè. The specific objectives are to determine the proportion of undernutrition and over nutrition among the WCA, to appreciate the adequacy of their diet to the nutritional recommendations and to investigate the interrelationship between their nutritional status and diet.

## Methods

**Study design:** a cross-sectional study with a descriptive and analytical purpose was conducted to assess the nutritional status and diet of women of childbearing age in the city of Comè in Benin.

**Study setting:** the city of Comè is located in the southeast of the Mono regional department, about sixty kilometers from Cotonou, the economic capital of Benin [14]. It includes thirty-eight (38) villages and neighbourhoods spread over the five (5) city districts which are: Comè, Agatogbo, Akodéha, Ouèdèmè-Pédah and Oumako [14]. The territory of the City of Comè covers a total area of 210 km^2^. According to the last national census, the population of the city was 79,989 in 2013 [15].

**Participants and eligibility criteria:** the study was conducted among women of childbearing age living in the city of Comè in Benin. Pregnant or lactating women were not included in the sample to avoid bias related to weight gain during these periods [16]. Of those included, those for whom the second 24-hour dietary recall has not been completed were excluded from the sample.

**Study variables and their measurement:** the variables considered for this study included those related to nutritional status (age, gender, weight, height), diet (number of meals per day, voluntary food restriction in the past 30 days, dietary diversity, total energy intake, macronutrient energy intake, protein intake, carbohydrate, fat and protein intake ratios, energy balance), socio-demographic characteristics (place of residence, age, marital status, education level, employment status, household size).

**Assessment of the nutritional status of WCA:** nutritional status was assessed by calculating and interpreting the Body Mass Index (BMI) after measuring the weight in kilograms and height in meters of each woman surveyed. For women aged 15 to 19 years, BMI was interpreted using World Health Organization (WHO) AnthoPlus software, which integrates the BMI curves for this age group. Thus, underweight corresponds to a BMI-for-age less than -2 Z-scores, normal nutritional status to a BMI-for-age ranging from -2 to +2 Z-scores, overweight to a BMI-for-age greater than +2 Z-scores but less than or equal to +3 Z-scores, and obesity to a BMI-for-age greater than +3 Z-scores according to the distribution of BMI-for-age of adolescents in the WHO reference population [17]. In women older than 19 years, underweight corresponds to a BMI less than <18.5 kg/m^2^, normal nutritional status to a BMI ranging from 18.5 kg/m^2^ to 24.99 kg/m^2^, overweight to a BMI ranging from 25 kg/m^2^ to 29.99 kg/m^2^, and obesity to a BMI greater than or equal to 30 Kg/m^2^ [18,19]. Weight was measured using a mechanical scale of the brand ILANIA® with a capacity of 120 Kilograms and an accuracy of 0.1 kg, and height was measured using a portable SECA® height gauge with a capacity of 2 meters and an accuracy of 0.1 cm.

**Assessment of nutritional intakes:** energy and macronutrient intakes were calculated from the food consumed by each woman surveyed. To reduce the biases inherent in any dietary data collection, two non-consecutive 24-hour dietary recalls (one on working days and one on weekends) were conducted. These recalls were conducted using the US Department of Agriculture's multiple-pass method [20]. To collect dietary data, a dietary kit was constructed to minimize bias in dietary intake estimates. It contained a set of images of local foods with corresponding portions and local measurements (bowls, spoons) known by the women surveyed. Some foods were weighed using a SECA® electronic diet scale with an accuracy of 0.1 g to estimate the amount consumed. Once the food data were collected, processing of these data followed the steps of (1) converting the collected food portions into grams, (2) finding the nutrient composition of each food consumed in the 2012 West African Food Composition Table, (3) calculating the nutrient intake of each WCA surveyed by multiplying the weight in grams of each food ingested by the amount of nutrients provided, (4) adjusting, normalizing the food intakes and estimating the usual intakes using the Multiple Source Methods (MSM) [21]. Once the nutrient intakes of interest were determined, they were compared to the Recommended Dietary Allowances (RDA) listed in the 2014 Benin Diet Manual for Adolescent Girls, Pregnant Women, Lactating Women and Newborns [22]. To assess the balance of the energy distribution of macronutrients in the diet, the standards for carbohydrates (55-75%), lipids (15-30%) and proteins (10-15%) retained by WHO and Food and Agriculture Organization (FAO) were used [23].

**Assessment of dietary diversity:** dietary diversity was assessed by calculating the Women Dietary Diversity Score (WDDS) [24]. This tool assesses dietary diversity in women based on the number of food groups consumed in the past 24 hours. The first 24-hour dietary recall administered to the surveyed women was used for this purpose. The nine (9) food groups are cereals, tubers and roots; dark green leafy vegetables; oils and fats; other fruits and vegetables; organ meats; meat and fish; eggs; legumes, nuts and seeds; and milk and milk products. If the food group was consumed 1 point is awarded. When it was not consumed no points are awarded (0). Because there is no established threshold in terms of the number of food groups to indicate adequate or insufficient dietary diversity, it is recommended to use the mean score or the distribution of scores [24]. In the present study, we opted for the mean score.

**Data collection:** the data for this study were collected in June 2017 by nutritionist-dietitians experienced in nutritional surveys. They were accompanied in the households by community health workers who had already participated in similar surveys. They have all been trained by the principal investigator to ensure proper selection of households, requesting the participation of WCA and mastery of the collection tools. A pre-test was done before the data collection to identify and correct the inadequacies of the tools. These tools included a structured questionnaire and a food data collection form. The interviewers were supervised by the principal investigator during the study. Each day before going, the scales were recalibrated to avoid measurement errors. Data were collected through face-to-face interviews. At the end of the day, the questionnaires and food data collection forms were checked to ensure that they were properly filled. As the collection progressed, the data were entered into excel files on the computer. Two data masks were designed on the basis of the collection tools. All excel were merged in a unique database for the analysis.

**Sample size:** the sample size was calculated by the SCHWARTZ formula:


n=εα2pqi2

with p the chronic energy deficit in Mono regional department in 2011 (7.5%) [25], εα^2^ the accepted risk of error (1.96^2^) and i the desired precision (3.7%). Adding 10% to cover non-response, the number of WCA to be surveyed was 215.

**Sampling method:** to have a representative sample, a two-stage random sampling was conducted. The sampling frame consisted of all the villages or neighbourhoods in the five (5) city districts of the city of Comè, numbered in ascending order with their respective population of women of childbearing age in 2017, drawn from the statistics of the Comè-Bopa-Grand-Popo-Houéyogbé health administrative area. The first stage consisted in selecting 50% of the districts and villages of the city, i.e. 19, by simple random draw. Thus, five (5) were chosen in the first arrondissement, four (4) in the second and fifth city districts, and three (3) in the third and fourth city districts. The number of women of childbearing age to be surveyed in each of these villages and districts was then determined in proportion to the population of WCA in each village and neighbourhood. The second stage involved selecting the WCA to be surveyed in each of the selected villages and neighbourhoods. The approach followed was to go to the center of the village or neighbourhood, throw a pen and choose the direction indicated by its lead, number all the concessions in that direction, choose by a simple random draw the first concession to visit, and then move from one to the next. Where there were multiple households in a building, one was selected by simple random draw. Similarly, when there were several women of childbearing age in a household, one was selected by simple random draw. When the village or neighbourhood was traversed without the desired number of women of childbearing age, it was necessary to return to the center of the village or neighbourhood, walk in the opposite direction from that originally chosen, and continue selecting households in the same way until the desired number was reached. If there were no women of childbearing age in the selected household, then move to the next concession.

**Ethical considerations:** the coordinating physician of the CBGH (Comè-Bopa-Grand-popo-Houéyogbé) health administrative area, the heads of the health centers, and the heads of the villages or neighbourhoods were informed of the survey in the selected villages and neighbourhoods. The purpose of the study as well as the terms of participation were presented to the WCA to obtain their voluntary, informed and oral consent. At any time during the survey, those who wished to do so we're able to withdraw. To guarantee anonymity and confidentiality, a code was assigned to each woman surveyed and marked on the collection tools.

**Funding:** the study was fully funded by the researchers.

## Results

**Sociodemographic characteristics of women of childbearing age:** the sociodemographic characteristics of the study population are presented in Table 1. A total of 215 women of childbearing age were surveyed, but four (04) were excluded because the second 24-hour dietary recall have not been performed. The median age was 32 (22; 40) years. Slightly more than half of the women surveyed lived in urban areas (52.1%). Slightly more than half of the women surveyed had attended school (24.2% primary school and 29.4% secondary school or higher). More than half (61.6%) were economically active. Christianity was the predominant religion (60.2%). The average size of the households in which these women of childbearing age resided was 5.7 ± 2.4 persons.

**Table 1:** sociodemographic characteristics of the women of childbearing age surveyed in Comè

Variables	n	%
**Place of residence**		
Urban	110	52.1
Rural	101	47.9
**Age**		
15-19	39	18.5
20-29	54	25.6
30-39	56	26.5
40-49	62	29.4
**Marital status**		
Married	146	69.2
Single	58	27.5
Divorced/Widowed	7	3.3
**Level of education**		
Non-literate	98	46.4
Primary	51	24.2
High school and up	62	29.4
**Professional situation**		
Employment	130	61.6
Student	41	19.4
Housekeeper	40	19.0
**Religion**		
Traditional	65	30.8
Christianity	127	60.2
Islam	7	3.3
Other	12	5.7

**Nutritional status of women of childbearing age:** among the women of childbearing age surveyed, the prevalence of underweight was 9.5%, overweight: 18% and obesity: 13.7%.

**Nutrition of women of childbearing age:** Table 2 presents the data on the diet of the WCA surveyed. Among the WCA surveyed, 20.4% said they ate less than three (03) meals a day and 9.9% said they had adopted a specific diet at least once in the last 30 days to lose weight. Regarding dietary diversity, the average WDDS was 4.6 ± 0.8. Among the WCA surveyed, 47.9% had a dietary diversity score below 5.

**Table 2:** dietary data of the women of childbearing age surveyed in Comè

Variables	n	%
**Number of meals per day**		
< 3	43	20.4
≥ 3*	168	79.6
**Recent diet for weight loss**		
Yes	21	9.9
No*	190	90.1
**Food diversity**		
Low (<5)	101	47.9
Good* (≥5)	110	52.1
**Normalized energy intake**		
< RDA	166	78.7
≥ RDA*	45	21.3
**Normalized protein intake**		
< RDA	25	11.8
≥ RDA*	186	88.2
**Standardized Protein Intake Ratio**		
< 10% of TCI	4	1.9
10-15% of TCI*	127	60.2
> 15% of TCI	80	37.9
**Standardized fat intake ratio**		
< 15% of TCI	203	96.2
15-30% of TCI*	8	3.8
> 30% of TCI	0	0.0
**Standardized carbohydrate intake ratio**		
< 55% of TCI	0	0.0
55-75% of TCI*	2	0.9
> 75% of TCI	209	99.1
**Energy balance**		
Imbalance	211	100
Balance	0	0.0

The mean total caloric intake (TCI) of the women surveyed was 2055.4 ± 634 Kcal. The mean protein intake was 68.7 ± 26 g. The mean fat intake was 37.8 ± 19 g and the mean carbohydrate intake was 395.4 ± 106 g. Compared with the recommended dietary allowance (RDA), 78.7% of the women of childbearing age surveyed had a low caloric intake and 11.8% had a low protein intake.

None of the surveyed WCA had a good energy distribution among macronutrients. Indeed, 99.1% had their carbohydrate intake ratio above the norm (55-75% of TCI), 96.2% had their fat ratio below the norm (15-30% of TCI) and 60.2% had the protein intake ratio within the norm (10-15% of TCI).

**Sociodemographic characteristics, dietary and nutritional status:** Table 3 presents the results of comparisons of women´s sociodemographic characteristics, dietary diversity and nutritional status. There were no significant associations of energy and protein intakes compared to RDA with sociodemographic characteristics, dietary diversity and nutritional status. From the comparison of sociodemographic characteristics with dietary diversity, a lower dietary diversity was observed among the oldest women (40-49 years), those with no education and those not employed with p<0.05. Dietary diversity was highest among the youngest women (15 to 19 years), those with education beyond primary school and being students with p<0.05.

**Table 3:** comparison between sociodemographic characteristics, dietary and nutritional status of the women of childbearing age surveyed in Comè

Variables	Food diversity				Nutritional status (BMI)					
	Good (≥5)		Low (<5)		Underweight		Normal		Excess weight	
	n	%	n	%	n	%	n	%	n	%
**Place of residence**										
Urban	62	56.4	48	43.6	12	10.9	63	57.3	35	31.8
Rural	48	47.5	53	52.5	8	7.9	61	60.4	32	31.7
**Age range**										
15-19	29	74.4	10	25.6**	2	5.1	36	92.3	1	2.6***
20-29	29	53.7	25	46.3	6	11.1	28	51.9	20	37.0
30-39	27	48.2	29	51.8	6	10.7	33	58.9	17	30.4
40-49	25	40.3	37	**59.7**	6	9.7	27	43.5	29	**46.8**
**Marital status**										
Married	69	47.3	77	52.7	14	9.6	77	52.7	55	37.7***
Single	41	70.7	17	29.3	4	6.9	44	75.9	10	17.2
Divorced/Widowed	0	0.0	7	100.0	2	28.6	3	42.9	2	28.6
**Level of education**										
Non-literate	41	41.8	57	58.2*	11	11.2	52	53.1	35	35.7**
Primary	30	58.8	21	41.2	7	13.7	22	43.1	22	43.1
High school and up	39	62.9	23	37.1	2	3.2	50	80.6	10	16.1
**Professional situation**										
Employment	63	48.5	67	51.5*	11	8.5	66	50.8	53	40.8***
Student	29	70.7	12	29.3	3	7.3	36	87.8	2	4.9
Housekeeper	18	45.0	22	**55.0**	6	15.0	22	55.0	12	30.0
**Religion**										
Traditional	27	41.5	38	58.5	9	13.8	38	58.5	18	27.7
Christianity	73	57.5	54	42.5	11	8.7	76	59.8	40	31.5
Islam	4	57.1	3	42.9	0	0.0	3	42.9	4	57.1
Other	6	50.0	6	50.0	0	0.0	7	58.3	5	41.7
**Household size**										
≤ 5	56	50.9	54	49.1	10	9.1	64	58.2	36	32.7
> 5	54	53.5	47	46.5	10	9.9	60	59.4	31	30.7
**Normalized energy intake**										
< RDA	87	52.4	79	47.6	13	7.8	99	59.6	54	32.5
≥ RDA*	23	51.1	22	48.9	7	15.6	25	55.6	13	28.9
**Normalized protein intake**										
< RDA	12	48.0	13	52.0	2	8.0	18	72.0	5	20.0
≥ RDA*	98	52.7	88	47.3	18	9.7	106	57.0	62	33.3
**Food diversity**										
Good	110	100.0	0	0.0	10	9.1	74	67.3	26	23.6*
Low	0	0.0	101	100.0	10	9.9	50	49.5	41	40.6

* p<0.05, **p<0.01, and ***p<0.001.

From the comparison of socio-demographic characteristics with nutritional status, we notice that the proportion of the excess weight (overweight and obesity) is the highest among the oldest women (40 to 49 years old), those who are married, those who have a primary level of education, those who have economic activity and those who have a low dietary diversity with p<0.05. For the underweight, its proportion is highest among divorced or widowed women and among those who are unemployed, but lowest among the youngest (15 to 19 years) and those with an education level beyond primary school with p<0.05.

## Discussion

This study assessed the nutritional status and diet of women of childbearing age residing in the city of Comè in Benin. The assessment of nutritional status revealed that 41.23% of the WCA surveyed had nutritional problems, not only because of deficiency (9.48% were underweight) but also because of excess weight (31.75% were overweight). Comparing these proportions with those reported by 2017-2018 Demographic and Health Survey (EDS-V) in the Mono regional department, of which Comè is one of the cities, some differences can be observed. Although overall the proportions of nutritional concerns among WCA are similar (39.6% in the Mono regional department), the prevalence of underweight in the present study is lower than in the department (13.5%) and that of overweight is higher than in the department (26.1%) [12]. These differences would be related to the disparities between cities in the population´s health status. In the present study, 47.9% of WCA had a low dietary diversity score (WDDS < 5). In a similar but earlier study conducted in Burkina Faso, 30% of the WCA had a score below 3 and less than 15% had a score above 5 [26]. Another study, conducted in Latin America, found 57.7% of women surveyed as having low dietary diversity [27]. In this study, dietary diversity was assessed using the Minimum Dietary Diversity Indicator for Women (MDD-W), which allows individual discrimination [24]. These studies, including the present one, show that for a large proportion of the WCA, dietary diversity, which is a guarantee of adequate micronutrient intake, is insufficient.

Compared to the RDA, the energy and protein intakes of the WCA in the Comè city are insufficient for 78.7% and 11.8% of them respectively. In a study in Morocco, the proportion of WCA with intakes below the RDA was 81.5% [28]. This study differs from the present by the fact that it was done only in an urban setting and lacked standardization of dietary intakes, probably due to the performance of a single 24-hour dietary recall. In China, a study of secular trends in energy and macronutrient intakes reported 47% and 48% of adult women did not meet the RDA for energy and protein [29]. In these different countries, as in Benin, the nutritional energy and protein intakes of WCA are inadequate for a large proportion of them.

Regarding the energy distribution among macronutrients, none of the surveyed WCA had an energy-balanced diet (ratios of the three macronutrients within the norms). The energy distribution was characterized by high carbohydrate intake (99.1% with a ratio >75% of TCI), low fat intake (96.2% with a ratio <15% of TCI), and adequate protein intake (60.2% with a ratio within 10-15% of TCI). This last result tends to confirm the comparison to RDA for protein. In the previously cited study conducted in China, the ratios of carbohydrate, fat and protein intakes were 51.5%, 35.8% and 12.5% respectively in adult women [29]. These ratios reflect a better energy distribution of macronutrients in the diet, although the authors of the study found an increase in fat intake that exceeded the upper limit (30%). The adequate protein ratio of WCA in the present study is a positive point, although the high predominance of carbohydrates and the low contribution of fat are of concern. Starchy foods are the staple foods of Beninese households and are consumed in large quantities; which explains the high contribution of carbohydrates to total energy intake. The city of Comè is adjacent to cities such as Grand-Popo and Kpomassè where fishing is practiced, so the availability of fish would favour protein consumption. As for fat consumption, it would be lower in this city due to its less urbanized nature compared to the large cities, which would slow down the nutritional transition characterized by a more sweet, salty and fatty diet.

Although this study allowed us to assess the nutritional status and diet of the WCA in the city of Comè, it has certain limitations. The first limitation is related to the lack of assessment of the level of physical activity of each woman surveyed, which would have allowed for a calculation of their individual energy needs for comparison with energy intakes. To assess the adequacy of energy intake to needs, the RDA established for WCA in Benin was used. The second limitation of the study is related to the assessment of dietary diversity of the WCA. The Minimum Dietary Diversity for Women (MDD-W) is a more recent tool than the WDDS, for which a minimum score has been established to discriminate more accurately between women with insufficient or sufficient dietary diversity [30]. Nevertheless, the WDDS remains a good proxy for predicting dietary micronutrient adequacy at the population level. The third limitation of the study is the lack of data on dietary habits in Benin, including food consumption frequencies, which would help refine the adjustment, normalization and estimation of usual intakes by the MSM method.

## Conclusion

This study assessed the nutritional status and diet of the WCA in the city of Comè in Benin. A significant proportion of the WCA is affected by nutritional problems due to deficiency and excess. Dietary diversity and nutritional intake are insufficient for a large proportion of them. These results reiterate the importance of developing nutritional interventions to improve their nutritional status and, by extension that of their children. Including indicators of the quality of the WCA´s diet in the national health information system and conducting periodically national food surveys to evaluate their real nutritional intakes will contribute to promoting better nutrition for them. This study is one of the few conducted in Benin on the diet of WCA. The information provided can help advance the knowledge and research on the nutritional status and diet of women of childbearing age in Benin, which is very relevant given the proportion of women with nutritional problems due to under- and over nutrition.

### What is known about this topic

- In developing countries, women of childbearing age are affected by undernutrition and over nutrition problems;
- The inadequate nutrition of women of childbearing age leads to poor pregnancy outcomes.

### What this study adds

- A significant proportion of women of childbearing age in the city of Comè in Benin is affected by nutritional problems due to deficiency and excess;
- Dietary diversity, energy distribution among macronutrients and nutritional intake are inadequate or insufficient for a large proportion of them.
